# Objective study validity diagnostics: a framework requiring pre-specified, empirical verification to increase trust in the reliability of real-world evidence

**DOI:** 10.1093/jamia/ocae317

**Published:** 2025-01-10

**Authors:** Mitchell M Conover, Patrick B Ryan, Yong Chen, Marc A Suchard, George Hripcsak, Martijn J Schuemie

**Affiliations:** Coordinating Center, Observational Health Data Science and Informatics, New York City, NY 10032, United States; Observational Health Data Analytics, Johnson & Johnson, Titusville, NJ 08560, United States; Coordinating Center, Observational Health Data Science and Informatics, New York City, NY 10032, United States; Observational Health Data Analytics, Johnson & Johnson, Titusville, NJ 08560, United States; Department of Biomedical Informatics, Columbia University Medical Center, New York City, NY 10032, United States; Coordinating Center, Observational Health Data Science and Informatics, New York City, NY 10032, United States; Department of Biostatistics, Epidemiology and Informatics (DBEI), The Perelman School of Medicine, University of Pennsylvania, Philadelphia, PA 19104, United States; Coordinating Center, Observational Health Data Science and Informatics, New York City, NY 10032, United States; Department of Biostatistics, University of California, Los Angeles, Los Angeles, CA 90095, United States; VA Informatics and Computing Infrastructure, US Department of Veterans Affairs, Salt Lake City, UT 20420, United States; Coordinating Center, Observational Health Data Science and Informatics, New York City, NY 10032, United States; Department of Biomedical Informatics, Columbia University Medical Center, New York City, NY 10032, United States; Coordinating Center, Observational Health Data Science and Informatics, New York City, NY 10032, United States; Observational Health Data Analytics, Johnson & Johnson, Titusville, NJ 08560, United States; Department of Biostatistics, University of California, Los Angeles, Los Angeles, CA 90095, United States

**Keywords:** observational study, research design, data interpretation, statistical, methods, causality

## Abstract

**Objective:**

Propose a framework to empirically evaluate and report validity of findings from observational studies using pre-specified objective diagnostics, increasing trust in real-world evidence (RWE).

**Materials and Methods:**

The framework employs objective diagnostic measures to assess the appropriateness of study designs, analytic assumptions, and threats to validity in generating reliable evidence addressing causal questions. Diagnostic evaluations should be interpreted before the unblinding of study results or, alternatively, only unblind results from analyses that pass pre-specified thresholds. We provide a conceptual overview of objective diagnostic measures and demonstrate their impact on the validity of RWE from a large-scale comparative new-user study of various antihypertensive medications. We evaluated expected absolute systematic error (EASE) before and after applying diagnostic thresholds, using a large set of negative control outcomes.

**Results:**

Applying objective diagnostics reduces bias and improves evidence reliability in observational studies. Among 11 716 analyses (EASE = 0.38), 13.9% met pre-specified diagnostic thresholds which reduced EASE to zero. Objective diagnostics provide a comprehensive and empirical set of tests that increase confidence when passed and raise doubts when failed.

**Discussion:**

The increasing use of real-world data presents a scientific opportunity; however, the complexity of the evidence generation process poses challenges for understanding study validity and trusting RWE. Deploying objective diagnostics is crucial to reducing bias and improving reliability in RWE generation. Under ideal conditions, multiple study designs pass diagnostics and generate consistent results, deepening understanding of causal relationships. Open-source, standardized programs can facilitate implementation of diagnostic analyses.

**Conclusion:**

Objective diagnostics are a valuable addition to the RWE generation process.

## Background and significance

Healthcare data such as electronic health records and administrative claims can be a valuable source of evidence on the effects of medical treatments. Recent advances in health information systems and data standards, and increased adoption of clinical systems has led to a growth in data networks and in observational studies worldwide. However, observational causal studies are often criticized for lack of reliability due to the potential for bias, particularly due to the perception that they are prone to type I errors (false positive findings). While concerns are frequently directed at confounding and misclassification bias, other factors that increase the number of false positives in observational literature include selection bias, p-hacking, and publication bias, which favors non-null over null findings.^1–5^

However, observational studies can produce reliable causal inferences when key assumptions are met. For example, in a comparative cohort study the assumption of no confounding can be tested by inspecting balance of measured covariates between target and comparator groups and inspecting negative control outcome distributions. As highlighted in multiple recent publications providing guidance on the reporting of observational research findings, assumptions that underlie observational studies should be tested and reported alongside results.^6–8^

In the current observational literature, reporting of evaluations of underlying study assumptions is severely limited. This makes interpreting the results of individual studies and understanding their reliability challenging. When such evaluations of assumptions do occur (eg, it is commonplace to report balance for a limited set of covariates), acceptable thresholds are rarely pre-specified, allowing the interpretation of these analyses to be influenced by the study findings. This invites p-hacking, where failure to meet underlying assumptions may be deemed tolerable when study results are sufficiently compelling.

Previously, we have proposed the Large-scale Evidence Generation and Evaluation across a Network of Databases (LEGEND) principles embodying a new paradigm for observational research aimed at addressing these issues.^9–11^ Key elements of LEGEND include generating evidence for many questions at once (eg, comparing all treatments for an indication for a large set of outcomes), using a standardized analytic design implementing current best practice, and disseminating this evidence without consideration of statistical significance. Following these principles prevents p-hacking and publication bias while minimizing bias due to observational study bias such as confounding. Here we propose to extend on this work by proposing a framework whereby objective diagnostic measures are used to evaluate and report the validity of study findings by either: (1) interpreting objective diagnostic results before unblinding study results or (2) only unblinding results from analyses for which all objective diagnostics pass *pre-specified* thresholds. Objective study validity diagnostics represent proactive, quantitative evaluations of the appropriateness of different study designs to yield reliable evidence addressing a causal question. Ideally, objective study validity diagnostics should be applied in sequence after passing empirical evaluations assessing whether data are fit-for-purpose (ie, data diagnostics) and empirical evaluations of measurement error in exposure and outcome definitions (cohort or phenotype diagnostics).^12–15^ Applying diagnostics in this order enables investigators to efficiently identify when studies are infeasible while providing clear, empirical explanations of infeasibility.

Ideally, multiple designs pass diagnostics and generate consistent results, strengthening our beliefs about causal relationships under study. We note that some study diagnostics are specific to certain study designs, while others can be employed across multiple study designs. In this paper, we will describe objective diagnostics that are suitable when conducting comparative cohort (CC) studies, though some can be employed across multiple study designs. To be clear, we assert these diagnostics only as a starting point to reflect current best practices. Further work is clearly needed to develop more informative and comprehensive diagnostics.

## Objective

We provide conceptual overviews of each objective diagnostic, the key assumption it tests, and considerations or references when pre-specifying diagnostic thresholds. As a thought experiment, we use negative control outcomes to conduct empirical evaluations of each diagnostic in the context of the LEGEND for Hypertension (LEGEND-HTN) cohort study, demonstrating their value to testing study design assumptions and serving as indicators of invalid findings.

## Methods

In Table 1 we provide a summary list of proposed objective diagnostics, the threats to validity they identify, a brief description of their calculation, and, when available, guidance on setting thresholds.

**Table 1.** Objective diagnostics, the threats to validity they identify, their calculation and guidance on setting thresholds.

Diagnostic	Threat to validity	Metric calculation	Threshold guidance
Minimum detectable relative risk	Misinterpreting wide effect estimates from grossly underpowered studies	Compute the minimum detectable relative risk (MDRR) metric and expected standard error (SE) for a given study population, using the actual observed sample size and number of outcomes (after analytic approaches have been applied).^17^$\mathrm{mdrr}=e^{\sqrt{\frac{{\left(Z_{\beta }+Z_{1-\frac{\alpha }{2}}\right)}^{2}}{\text{total Events}*P_{A}*P_{B}}}}$	We propose MDRR < 10, although there is debate whether power calculations have utility in studies using pre-existing observational data.^18–21^
Empirical equipoise	Confounding^24^ Non-positivity^23^	$\ln(\frac{F}{\left(1-F\right)}=\ln\left(\frac{S}{1-S}\right)-\ln(\frac{P}{1-P})$ *F* = preference score *S* = propensity score for receiving target *P* = Fraction of people receiving target	0.3 ≤ F ≤ 0.7 in more than half of patients^24^
Covariate balance maximum standardized difference of means (SDM)	Confounding bias^26–28^	The SDM compares the proportion or mean of exposed and unexposed, scaled to the pooled standardized deviation. The maximum SDM is the largest SDM measured across all observed baseline variables. $\mathrm{SDM}=\frac{{(\bar{x}}_{T}-{\bar{x}}_{C})}{\sqrt{\frac{s_{T}^{2}+s_{C}^{2}}{2}}}$ for continuous variables SDM=(p^T-p^C)p^T1-p^T+p^C(1-p^C)2 for dichotomous variables T = target, C = comparator	SDM_max_ > 0.10 conventionally interpreted to indicate the presence of confounding bias based on Austin et al. heuristic.^26–29^
Generalizability maximum SDM	Selection bias^31^	Same calculation as covariate balance SDM, comparing analytic vs target population	*SDM*_max_ < 0.25 suggested as a rule of thumb to indicate that the population is “like a random sample”^31^^,^^32^
Expected Absolute Systematic Error (EASE)	Systematic error (selection, confounding, misclassification bias)^1^	$\mathrm{EASE}=\mathrm{average}\left(\left|{\ln(HR}_{\mathrm{estimate}}\right)-\ln\left({HR}_{\mathrm{truth}}\right)\right|)$ across negative control outcome studies	A current rule of thumb is EASE < 0.25.

### Minimum detectable relative risk

Because for any given observational study the sample size is fixed (we cannot choose to enroll more subjects), we prefer to express statistical power as the minimum detectable relative risk (MDRR) given the sample size and requiring 80% power (20% type 2 error) (eg, MDRR = 10 implies that you have sufficient data to detect a relative risk of 10 or larger).^16^^,^^17^ The utility of power calculations when using pre-existing observational data is debated, given that even under-powered observational effect estimates may still meaningfully contribute to informative meta-analyses.^18–21^ Indeed, Hernan et al. stated “the solution to observational analyses with imprecise effect estimates is not avoiding observational analyses with imprecise estimates, but rather encouraging the conduct of many observational analyses.”^18^ We argue to require some minimum power threshold (eg, MDRR > 10), given that studies that are grossly underpowered produce unstable estimates with wide confidence intervals that people struggle to interpret correctly.

### Empirical equipoise

Equipoise refers to clinical uncertainty and variation with respect to treatment choices made by patients and clinicians. *Empirical* equipoise measures this preference by using observed data on treatment choices as a proxy for true equipoise. This can be visually assessed by examining the overlap in propensity or preference score distributions, making it a critical tool for evaluating the assumption of positivity (ie, sufficient variation in exposure across study exposures and covariates).^22–25^ In simpler terms, empirical equipoise determines if there is a sufficiently large group of similar patients (based on observed covariates) for whom there is genuine uncertainty about which treatment they will receive.

Empirical equipoise can be estimated by transforming propensity score estimates into preference scores which, like propensity scores, are bound by 0 and 1 but, unlike propensity scores, are scaled to the prevalence of the exposures in the population.^24^ Patients with preference scores between 0.3 and 0.7 are said to be in empirical equipoise and Walker et al. suggests that study findings are most likely to be accurate when at least 50% of patients were in empirical equipoise. It is important to note that the estimates of empirical equipoise are a function of the underlying propensity score model it is based on. A propensity score model that leaves out critical explanatory variables that determine treatment assignment may indicate that empirical equipoise is sufficient when it is not.

### Covariate balance: maximum SDM

The maximum standardized difference of means (SDM) is a measure of covariate imbalance which serves as an indicator of potential confounding bias in comparative studies. In comparative studies, the SDM statistic is used to compare patients in the target and comparator cohorts with respect to baseline characteristics, typically assessed over some time window on or before index. Specifically, the statistic compares proportions or mean scaled to the pooled standardized deviation. The maximum SDM is the largest SDM measured across all observed baseline variables (which should include but should not be limited to those variables selected for use in the propensity score model). Austin et al. provided an informal cut-point to determine analytic scenarios where covariate imbalance implied a potential threat to the validity of effect estimates due to confounding bias (the largest SDM across measured covariates > 0.10).^26–29^ However, this cut-point was defined when typical observational studies measured and adjusted for small sets of manually curated covariates. Whether this threshold is still appropriate in the context of modern studies that control for large, highly dimensional covariate sets is a topic of ongoing research.^30^

### Generalizability

In observational studies it is often necessary to alter the study population to ensure correct causal estimation. For example, propensity score matching may remove people at the extremes of the distribution who have no match, or weighting may place more emphasis on some parts of the population. For this reason, the analytic cohort, the cohort used in the final analysis, may have a different composition than the target cohort, the cohort for which we wish to answer the question. We can compare clinical characteristics of these two cohorts using the SDM, with large differences in characteristics indicating questionable generalizability.^31^ It is important to note that the generalizability and attrition diagnostics applied here provide a partial view of external validity and are not intended to inform potentially meaningful differences between patients captured in databases versus the patients in the population we seek to generalize inferences to. A diagnostic threshold of *SDM*_max_ < 0.25 has been suggested as a rule of thumb, which has been partially supported by simulation results.^31^^,^^32^

### Expected absolute systematic error

Negative controls are a common tool in experimental and non-experimental research used to detect error. In this context, a negative control refers to an exposure-outcome pair where it is known or expected *a priori* that the true causal effect is null.^1^^,^^7^^,^^33–35^ Applying our study design and analysis to a negative control outcome allows us to confirm that the design produces the expected answer; with deviation from the true null effect indicating the presence of bias. For example, in a study estimating the relative effect of two antihypertensive treatments on acute myocardial infarction, a negative control experiment might apply the same comparison and study design to a negative control outcome of ingrowing nail. Using a large set of negative controls allows us to understand and quantify the distribution of these errors, providing a holistic evaluation of systematic error.^9–11^^,^^36^^,^^37^ The distribution of systematic errors, quantified on a logarithmic scale, is referred to as the empirical null distribution.

The empirical null distribution serves two purposes: as a diagnostic measure of study validity and as a quantifiable value that can be used to adjust or “calibrate” effect estimates, confidence intervals and *P*-values. While calibration can be used to account for known systematic errors and has been demonstrated to reduce bias in effect estimates, an empirical null distribution that indicates a large degree of systematic error should be interpreted as an indicator to redesign the study or to forego unblinding to and interpreting effect estimates if no redesign is possible. We summarize the empirical null distribution in a single metric, the expected absolute systematic error (EASE), by integrating over the absolute value of this distribution. We propose a threshold of EASE < 0.25. When EASE = 0.25 and systematic error is centered on 0, a true relative risk of 1 has a 95% probability of appearing to be anywhere between 0.54 and 1.85 due to systematic error.

### Empirical evaluation of diagnostics for comparative cohort design using the LEGEND-HTN study

To demonstrate the value of each objective diagnostic as an indicator of potentially invalid study results, we conducted a large-scale evaluation estimating effects corresponding to a large set of analyses using negative control outcomes. These analyses derive from the LEGEND-HTN study and included on-treatment comparisons of the effect of various monotherapy antihypertensive treatments (Table S1) on 11 716 negative control exposure-comparator-outcome triplets.^9^^,^^11^ A brief methodological overview of the LEGEND-HTN study design and statistical analyses is provided in Text S1.

Negative controls were deemed suitable if (1) neither target nor comparator drug has the outcome on the label and (2) no other drug in the same class has the outcome on the label. This new set of negative controls, developed specifically for this evaluation of the value of applying objective diagnostics, differed from the set of negative controls used in the LEGEND-HTN study itself. The original set of negative controls was defined as a generic set of outcomes for all antihypertensives, requiring no evidence in literature, spontaneous reports, and product labels that any antihypertensives might cause the outcome. As such, the LEGEND negative controls were more certain to be negative, because more evidence sources were consulted. We provide more detailed description of the methods used to select LEGEND negative control outcomes in Text S1. The use of a broader set of negative controls for this diagnostic evaluation was motivated by a need to (1) demonstrate the value of objective diagnostics restricting on EASE in the original LEGEND study, using the LEGEND negative control set, and (2) to expand the list of negative control experiments which yields more statistical information for this analysis. Large-scale propensity score (LSPS) adjustment (stratification and variable-ratio matching) was used to control confounding and the analysis was conducted across a global network of nine databases (six administrative claims databases and three electronic health record databases).^38^ The claims databases were: (1) Merative MarketScan Commercial Claims and Encounters (CCAE, US employer-based private payer—patient ages ≤ 65), (2) Optum ClinFormatics (Optum, US private-payer—primarily ≤ 65), (3) Merative MarketScan Medicare Supplemental Beneficiaries (MDCR, US retirees—65+), (4) Merative MarketScan Multi-state Medicaid (MDCD, US Medicaid enrollees—all ages), (5) Japan Medical Data Center (JMDC, Japan private-payer—18-65), and (6) Korea National Health Insurance Service/National Sample Cohort (NHIS/NSC, South Korea—all ages); the EHRs are: (1) Optum Pan-Therapeutic (PanTher, US health systems—all ages), (2) IMS/IQVIA Disease Analyzer Germany (IMSG, German ambulatory-care—all ages), and (3) Columbia University Medical Center (CUMC, US academic health system—all ages). All data partners obtained either Institutional Review Board approval or exemption before participating. More detailed database descriptions are provided in the Supplementary Materials (Text S2).

For each negative control analysis, we calculated the above-described diagnostics, and then computed the distribution of diagnostics statistics across the full set of studies. We also plotted the negative control distribution (ie, the full set of effect estimates plotted against their standard errors) and evaluated the EASE statistic among the full set of studies and a subset of studies meeting commonly accepted thresholds applied for each diagnostic (MDRR ≤ 10, equipoise ≥ 0.50, covariate balance SDM < 0.10, generalizability SDM ≤ 0.25, systematic error (EASE) ≤ 0.25). The change in EASE statistic (EASE_Δ_) quantifies the impact on systematic error when applying the various objective diagnostic approaches to blind invalid study findings.

In addition to negative control outcomes, we also applied diagnostic thresholds to the full set of LEGEND-HTN results (Table S2) and demonstrate the impact on the distribution of effect estimates. All diagnostic evaluations were executed using standard, open-source R packages from the Observational Health Data Sciences and Informatics (OHDSI) community’s Health Data Analytics-to-Evidence Suite (HADES).^39^ The application of objective study validity diagnostics is facilitated by the standardization of data sources using the Observational Medical Outcomes Partnership (OMOP) Common Data Model (CDM) and standard analytic approaches applied using the HADES software, which are described in more detail in the Supplementary Materials (Text S3). However, the application of objective study validity diagnostics should be considered for all studies and may be even more useful when interpreting studies that do not use standardized, quality-controlled programs.

## Results

In Table 2, we provide summary statistics (log-HR_µ_, EASE and percent of CIs excluding the null) describing evaluations of systematic error among negative control studies restricted on various objective diagnostic thresholds. We present statistic EASE_Δ_ to quantify the change in systematic error after applying each diagnostic threshold. The proportion of studies satisfying diagnostic criteria was similar for the full set of LEGEND-HTN outcomes and the negative control outcomes. Figures providing detailed results for each separate diagnostic criterion are provided in Figures S1-S5 and results of the analysis applying the multiple different commonly accepted diagnostic thresholds in aggregate are provided in Figure S6.

**Table 2.** Summary statistics describing evaluations of systematic error among negative control studies (ie, using empirical null distribution), after restricting on various objective diagnostic thresholds.

	LEGEND studies	LEGEND negative control studies				
Diagnostic threshold(s)	*N* (% satisfied)	*N* (% satisfied)	log-HR_µ_ (SD)^a^	EASE	EASE_Δ_	CIs excl. null (%)
None	471 321 (100.0%)	11 716 (100.0%)	0.00 (0.48)	0.38	–	15.2%
All^b^	54 358 (11.5%)	1633 (13.9%)	0.00 (0.00)	0.00	−0.38	3.9%
MDRR < 10	447 445 (94.9%)	11 233 (95.9%)	0.00 (0.48)	0.38	0.00	15.7%
Equipoise > 0.5	136 405 (28.9%)	2792 (23.8%)	0.00 (0.02)	0.02	−0.36	4.7%
Equipoise > 0.1	413 489 (87.7%)	10 010 (85.4%)	0.00 (0.41)	0.33	−0.05	13.5%
Covariate balance SDM < 0.1	204 758 (43.4%)	4923 (42.0%)	0.00 (0.35)	0.28	−0.10	11.0%
Generalizability SDM < 0.25	203 986 (43.3%)	4942 (42.2%)	0.03 (0.47)	0.37	−0.01	13.9%
EASE < 0.25	394 953 (83.8%)	9718 (82.9%)	0.00 (0.44)	0.35	−0.03^c^	14.3%

a The log-HR_µ_ (SD) statistic presented here refers to the mean hazard ratio on the logarithmic scale among all negative control studies (ie, the empirical null distribution).

b MDRR ≤ 10, equipoise ≥ 0.50, covariate balance SDM < 0.10, generalizability SDM ≤ 0.25, systematic error (EASE) ≤ 0.25.

c As described in the methods section, the diagnostic EASE threshold was applied to a generic set of negative control outcomes for all antihypertensives, requiring no evidence in literature, spontaneous reports, and product labels that any antihypertensives might cause the outcome. The evaluation of EASE_Δ_ reflects a more inclusive set of negative controls which satisfied the following criteria: (1) neither target nor comparator drug has the outcome on the label and (2) no other drug in the same class has the outcome on the label.

Before applying any diagnostic thresholds, estimates from the full set of negative control analyses were evenly dispersed (HR_µ_=0.stu00, SD = 0.48). The negative controls exhibited a high degree of systematic error, as indicated by the high EASE statistic (EASE = 0.38), which corresponded to 15.2% of the estimates having confidence intervals excluding the null.

**Figure 1.**
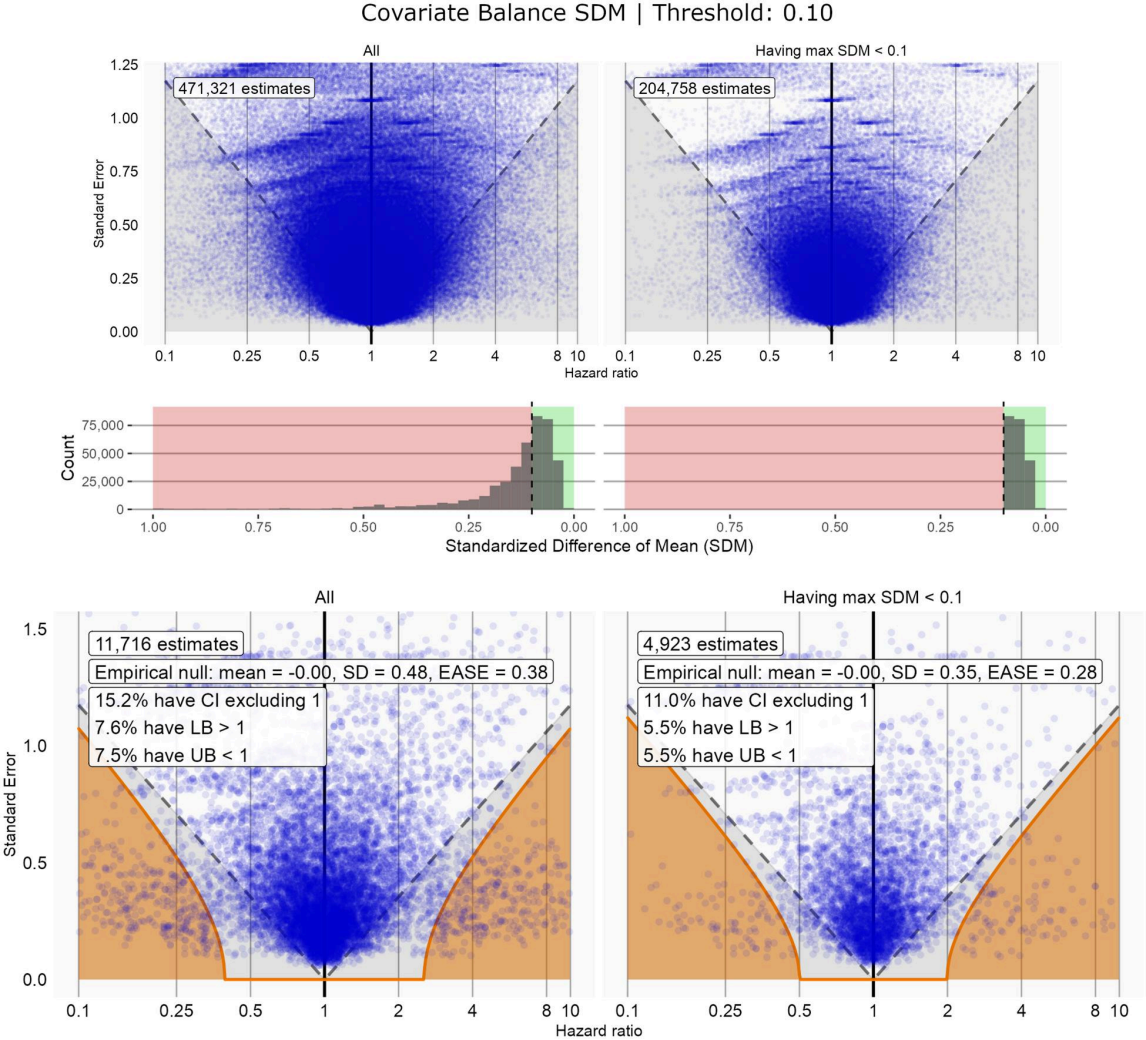
Top panel: effect estimates plotted against standard errors for the full set of LEGEND-HTN analyses (left) and those with covariate-balance SDM < 0.1 (right). Middle panel: the distribution of the covariate-balance standardized difference of means (SDM) for all negative control analyses (left) and those with covariate-balance SDM < 0.1 (right). Bottom panel: the empirical null distribution for all negative control analyses (left) and those with covariate-balance SDM < 0.1 (right).

Detailed results of the analysis of the covariate balance diagnostic analysis are presented in Figure 1, using the threshold SDM < 0.10. We have chosen to foreground the covariate balance diagnostic because it is one of the most commonly used in observational research; however, figures describing other objective diagnostics are included in the Supplementary Materials and are described below. The covariate balance diagnostic was restrictive. As shown, among the full set of 11 716 negative control analyses, only 4923 (42.0%) passed. After restricting to negative control analyses that passed the diagnostic, estimates remained evenly dispersed (HR_µ_=0.00) and the EASE statistic was reduced to EASE = 0.28 (Table 2, Figure 1). Confidence interval coverage also improved, with 11.0% of estimates having confidence intervals excluding the null (aligning more closely with statistical expectation compared to the unrestricted analysis).

In the top and bottom panels of Figure 1, estimates appearing below the gray dashed lines have traditional confidence intervals that exclude the null. In the bottom panel, estimates shown in the orange area represent a subset which have calibrated confidence intervals exclude the null. Comparing the lower-left and lower-right panel in Figure 1 demonstrates that restricting on the covariate balance SDM diagnostic reduces the number of negative control estimates with confidence intervals that exclude the null (as noted by the decrease in the CI excluding 1 statistic) and more closely centers the distribution around one (as noted by the decrease in the EASE statistic).

The MDRR threshold (MDRR < 10) was relatively unimpactful due to the vast majority (95.9%) of negative control analyses satisfying the criteria (Figure S1). The same was the case for the diagnostics applying an equipoise threshold >0.1 (Figure S3) and an EASE threshold <0.25 (Figure S5), which were satisfied by 85.4% and 82.9% of negative control analyses respectively. The generalizability SDM diagnostic threshold (SDM < 0.25) was fairly restrictive (satisfied by 42.2% of analyses), however had little impact on systematic error (EASE_Δ_ = −0.01) (Figure S4). (This may be an expected finding given that, in the context of our negative control outcome evaluation, we expect no effect modification as we would when studying non-null effects.) Last, the diagnostic requiring >50% of patients be in equipoise was both the most restrictive (satisfied by only 23.8% of analyses), but also the most impactful, reducing the systematic error to near-zero (EASE_Δ_ = −0.36) (Figure S2).

In the analysis restricting to negative control analyses that satisfied all specified objective diagnostics (set to commonly accepted thresholds), the EASE statistic was reduced to zero and 3.9% of negative control estimates had 95% confidence intervals excluding the null (Table 2, Figure S6). When evaluated individually, the equipoise diagnostic (equipoise > 0.5) was the most impactful (EASE_Δ_ = −0.36); however, the combined approach applying multiple diagnostics further reduced EASE, indicating the complementary role the diagnostics serve when applied alongside each other. Finally, we observed some interaction between the covariate balance and generalizability diagnostics which had small impacts on their own but had more substantial impact on EASE when applied together (data not presented).

## Discussion

Increasing use of real-world data presents a substantial scientific opportunity; however, the real-world evidence (RWE) generation process is complex due to a wide range of investigator design choices and limited empirical evidence confirming that those choices improve study validity. The LEGEND principles introduced a standardized framework for generating evidence intended to increase reliability of RWE. Still, concerns of residual bias remain. Objective diagnostics provide a pre-specification framework to empirically evaluate the reliability of evidence before unblinding results, which can be applied in the context of any observational study. In this study, we demonstrate that objective diagnostics can reduce residual bias and provide a basis for applying to individual studies and large-scale evidence systems alike. Furthermore, these diagnostic analyses can be implemented using open-source, standardized programs.^39^

Each diagnostic is designed to identify a threat to study validity. When applied together the suite of objective diagnostics provide a comprehensive set of tests which improve confidence when passed and cast doubt when failed. Furthermore, pre-specification of decision thresholds for each diagnostic reduces the risk introduced by post hoc rationalization of diagnostic failures or violations of key assumptions. Unblinding only results that pass pre-specified thresholds prevents investigators from viewing and misinterpreting potentially spurious results. Objective diagnostics can be used to set clear objective reliability standards that can be easily communicated in a protocol prior to study execution and verified through to results dissemination.

When we broadly apply diagnostic thresholds across evidence systems (eg, covariate balance SDM > 0.10 being widely interpreted to indicate a problematic potential for confounding bias) there are two concerns. Setting the threshold too liberally risks unblinding and misinterpreting unreliable study results while setting it too conservatively risks masking ourselves to potentially valid, important findings. However, by using objective diagnostics to blind unreliable findings from interpretation, the potential trade-off between the frequency of false positives and false negative findings becomes a trade-off between false positive and “inestimable” findings. Through blinding unreliable estimates, objective diagnostics can reduce the rate of both false positives and false negatives simultaneously.

Diagnostics are only as good as their inputs. A comprehensive assessment of all covariates is important to providing informative diagnostics, regardless of whether smaller sets of covariates were elected appropriate for modeling. Small, manually curated sets of covariates provide limited evaluations of important determinants of reliability including generalizability, covariate balance, and equipoise. Similarly, studies should use a sufficiently large set of negative control outcomes to reliably estimate the distribution systematic error.^36^

There are several important limitations to this work. First, our use of negative control outcomes to conduct our evaluation prevents us from quantifying the frequency of false negative findings (given that all effects are truly negative). However, diagnostic evaluations would clearly decrease false negatives given the large proportion of studies that are re-classified as “inestimable.” Second, the use of negative control outcomes likely skews our evaluation of the generalizability SDM diagnostic. Given that we do not expect modification of (null) negative control effects between the target and analytic cohort, we would not expect differences in the two population to meaningfully impact effect estimates. Third, we only explored a limited set of diagnostic thresholds. In the case of the MDRR diagnostic, the threshold we applied (MDRR < 10) excluded very few negative control studies (in part because the LEGEND-HTN study only studied target and comparator treatments that met minimum size requirements). Applying a more meaningfully restrictive threshold may yield different findings. Fourth, these analyses provide only limited information on the interaction between various diagnostic assessments and their relative value. For example, the EASE_Δ_ statistics in Table 2 indicate that after applying the equipoise diagnostic, other diagnostics have little additional impact on systematic error. However, the equipoise diagnostic is highly restrictive, so relaxing that threshold while applying some combination of other diagnostics may still be preferable. Finally, these analyses explore a small set of potential diagnostic evaluations and alternative diagnostics may be better suited. For example assessing whether SDMs *significantly* exceed the threshold of 0.1 may be better suited in scenarios where sample size is low and evaluating covariate balance using the post-matching C-statistic may be better suited to identify scenarios where a large number of minorly imbalanced but directionally persistent confounders produce meaningful bias.^30^^,^^40^ Further work is needed developing and evaluating the performance of objective diagnostics.

While diagnostics always provide insight, we note that applying a stringent diagnostic threshold to blind results may not always be necessary. Even in cases where relaxed thresholds are deemed acceptable, it is still valuable to pre-specify them. For example, the generalizability SDM diagnostic tells us when our analytic population meaningfully differs from our target population. However, as pointed out by Rothman (2014), valid generalizations of causal inferences can be derived from non-representative samples.^41^ Also notable, diagnostic thresholds may be pre-specified differently when meta-analyzing results (eg, generated by multiple studies conducted across a distributed data network). For example, we may remain blinded to individual database results due to failures on diagnostics like the MDRR, equipoise, or generalizability SDM but still choose to include those results in meta-analysis. Future work exploring the role of objective diagnostics in improving the validity and reliability of meta-analyses would be informative.

In closing, standardization of programs and evidence generation enable a comprehensive and rigorous inspection of validity and reliability. Through standardization and open-source development, we hope to socialize a common set of diagnostic evaluations, which can be easily deployed by investigators and broadly understood by the field, reducing the cognitive burden on the consumers of observational research and improving access to reliable medical evidence. Here we demonstrate the clear value of applying objective, empirical, and pre-specified diagnostic criteria when generating and interpreting evidence from observational research. While work remains, we believe that these diagnostics are crucial for evaluating and communicating the reliability of evidence generated by observational studies.

## Supplementary Material

ocae317_Supplementary_Data

## Data Availability

All databases used in this study are standard, secondary healthcare databases available for public licensure.
