# Supplementary material for: Objective study validity diagnostics: a framework requiring pre-specified, empirical verification to increase trust in the reliability of real-world evidence
Source: J Am Med Inform Assoc. 2025 Jan 10;32(3):518–25. doi: 10.1093/jamia/ocae317 (PMC11833483; doi:10.1093/jamia/ocae317)
Supplement: ocae317_Supplementary_Data [file ocae317_supplementary_data.docx]

**Supplemental Materials**

**Table S1.** Hypertension treatments included in this study

| **Ingredient** | | **Class** | **Major class** |
| --- | --- | --- | --- |
| Benazepril | Moexipril | ACE inhibitors | Angiotensin converting enzyme (ACE) inhibitors |
| Captopril | Perindopril |  |  |
| Enalapril | Quinapril |  |  |
| Fosinopril | Ramipril |  |  |
| Lisinopril | Trandolapril |  |  |
| Doxazosin | Terazosin | Alpha-1 blockers | Alpha-1 blockers |
| Prazosin |  |  |  |
| Azilsartan | Losartan | Angiotensin receptor blockers | Angiotensin receptor blockers |
| Candesartan | Olmesartan |  |  |
| Eprosartan | Telmisartan |  |  |
| Irbesartan | Valsartan |  |  |
| Atenolol | Bisoprolol | BB cardioselective | Beta-blockers (BB) |
| Betaxolol | Metoprolol |  |  |
| Nebivolol |  | BB cardioselective and vasodilatory |  |
| Carvedilol | Labetalol | BB combined alpha and beta receptor |  |
| Acebutolol |  | BB intrinsic sympathomimetic activity |  |
| Penbutolol | Pindolol |  |  |
| Nadolol | Propranolol | BB non-cardioselective |  |
| Amlodipine | Nicardipine | Dihydropyridine CCB (dCCB) | Calcium Channel Blockers (CCB) |
| Felodipine | Nifedipine |  |  |
| Isradipine | Nisoldipine |  |  |
| Diltiazem | Verapamil | Nondihydropyridine CCB (ndCCB) |  |
| Hydralazine | Minoxidil | Direct vasodilators | Direct vasodilators |
| Eplerenone | Spironolactone | Aldosterone antagonist diuretics | Diuretics |
| Bumetanide | Torsemide | Loop diuretics |  |
| Furosemide |  |  |  |
| Amiloride | Triamterene | Potassium sparing diuretics |  |
| Chlorthalidone | Indapamide | Thiazide or thiazide-like diuretics (THZ) |  |
| Hydrochlorothiazide | Metolazone |  |  |
| Aliskiren | Guanfacine |  |  |
| Clonidine | Methyldopa |  |  |

Abbreviations: ACE, angiotensin converting enzyme; BB, beta blocker; CCB, Calcium Channel Blockers; dCCB, Dihydropyridine CCB; ndCCB, Nondihydropyridine CCB; THZ, thiazide.

**Table S2.** Health outcomes of interest

| Abdominal pain | Dementia | Ischemic stroke |
| --- | --- | --- |
| Abnormal weight gain | Depression | Malignant neoplasm |
| Abnormal weight loss | Diarrhea | Measured renal dysfunction |
| Acute myocardial infarction | End stage renal disease | Nausea |
| Acute pancreatitis | Fall | Neutropenia or agranulocytosis |
| Acute renal failure | Gastrointestinal bleeding | Rash |
| All-cause mortality | Gout | Rhabdomyolysis |
| Anaphylactoid reaction | Headache | Stroke |
| Anemia | Heart failure | Sudden cardiac death |
| Angioedema | Hemorrhagic stroke | Syncope |
| Anxiety | Hepatic failure | Thrombocytopenia |
| Bradycardia | Hospitalization with heart failure | Transient ischemic attack |
| Cardiac arrhythmia | Hospitalization with preinfarction syndrome | Type 2 diabetes mellitus |
| Cardiovascular event | Hyperkalemia | Vasculitis |
| Cardiovascular-related mortality | Hypokalemia | Venous thromboembolic events |
| Chest pain or angina | Hypomagnesemia | Vertigo |
| Chronic kidney disease | Hyponatremia | Vomiting |
| Cough | Hypotension |  |
| Decreased libido | Impotence |  |

**Text S1.** Methodological overview of LEGEND-HTN study design and statistical analyses

LEGEND-HTN is a retrospective, comparative new-user cohort study which included patients initiating monotherapy (**Table S1**) with one of five hypertension drug classes: 1) thiazide or thiazide-like diuretics (THZ), 2) angiotensin converting-enzyme inhibitors (ACEi), 3) angiotensin receptor blockers (ARB), 4) dihydropyridine calcium channel blockers (dCCB), or 5) non-dihydropyridine calcium channel blockers (ndCCB). Ten pairwise comparisons were conducted across these drug classes.

For each comparison, the LEGEND-HTN study assessed 55 outcomes (nine effectiveness and 46 safety), listed in **Table S2**. Patients with a history of the outcome were excluded and incident outcomes were identified using on-treatment and intent-to-treat time-at-risk definitions. On-treatment time was based on prescription data, assuming discontinuation if a gap of >30 days occurred between prescriptions.

Measured confounding was adjusted using large-scale propensity score adjustment. Propensity scores were constructed for each comparison using a data-driven, regularized regression procedure to select from among thousands of features those that are most predictive of treatment.^1^ Patients were stratified or variable-ratio matched by propensity score (conditioned on the strata/matched set, yielding the conditional likelihood) to achieve covariate balance. Adjusted populations were analyzed with Cox proportional hazards models, and hazard ratios were meta-analyzed across databases using a random-effects model.^2^

Negative and synthetic positive controls were employed to calibrate hazard ratios and 95% confidence intervals.^3–5^ Additionally, three study validity diagnostics were reported: minimum detectable risk ratio, empirical equipoise, and covariate balance (maximum standardized mean difference).

LEGEND-HTN selected negative controls using criteria adapted from Voss et al. ^6^ Conditions were included as negative controls if they satisfied the following criteria:

- No Medline abstract suggested a drug-condition association based on MeSH terms.^7^
- The drug-condition pair was absent from US product labels under "Adverse Drug Reactions" or "Postmarketing" sections.^8^
- No US spontaneous reports linked the drug-condition pair as an adverse event.^9,10^
- The OMOP vocabulary does not identify the condition as an indication for the drug
- Concepts were usable (e.g., not overly broad, suggestive of adverse events, or pregnancy-related).
- The exact condition concept appeared in patient-level data.

Lastly, concepts for negative controls were optimized using the OMOP vocabulary and manual review was used to remove drug-condition pairs with potential causal relationships that were not eliminated by the above criteria. This process resulted in a list of 76 negative controls:

**Table S3.** LEGEND-HTN negative control outcomes

| **Condition** |
| --- |
| Abnormal cervical smear |
| Abnormal pupil |
| Abrasion and/or friction burn of trunk without infection |
| Absence of breast |
| Absent kidney |
| Acid reflux |
| Acquired hallux valgus |
| Acquired keratoderma |
| Acquired trigger finger |
| Acute conjunctivitis |
| Amputated foot |
| Anal and rectal polyp |
| Burn of forearm |
| Calcaneal spur |
| Cannabis abuse |
| Cervical somatic dysfunction |
| Changes in skin texture |
| Chondromalacia of patella |
| Cocaine abuse |
| Colostomy present |
| Complication due to Crohn’s disease |
| Contact dermatitis |
| Contusion of knee |
| Crohn’s disease |
| Derangement of knee |
| Difficulty sleeping |
| Disproportion of reconstructed breast |
| Effects of hunger |
| Endometriosis |
| Epidermoid cyst |
| Feces contents abnormal |
| Foreign body in orifice |
| Ganglion cyst |
| Genetic predisposition |
| Hammer toe |
| Hereditary thrombophilia |
| Herpes zoster without complication |
| High risk sexual behavior |
| Homocystinuria |
| Human papilloma virus infection |
| Ileostomy present |
| Impacted cerumen |
| Impingement syndrome of shoulder region |
| Ingrowing nail |
| Injury of knee |
| Irregular periods |
| Kwashiorkor |
| Late effect of contusion |
| Late effect of motor vehicle accident |
| Leukorrhea |
| Macular drusen |
| Melena |
| Nicotine dependence |
| Noise effects on inner ear |
| Non-toxic multinodular goiter |
| Nonspecific tuberculin test reaction |
| Onychomycosis due to dermatophyte |
| Opioid abuse |
| Passing flatus |
| Postviral fatigue syndrome |
| Presbyopia |
| Problem related to lifestyle |
| Psychalgia |
| Ptotic breast |
| Regular astigmatism |
| Senile hyperkeratosis |
| Somatic dysfunction of lumbar region |
| Splinter of face, without major open wound |
| Sprain of ankle |
| Strain of rotator cuff capsule |
| Tear film insufficiency |
| Tobacco dependence syndrome |
| Vaginitis and vulvovaginitis |
| Verruca vulgaris |
| Wrist joint pain |
| Wristdrop |

**References:**

1. Tian Y, Schuemie MJ, Suchard MA. Evaluating large-scale propensity score performance through real-world and synthetic data experiments. *International Journal of Epidemiology*. 2018;47(6):2005-2014. doi:10.1093/ije/dyy120

2. DerSimonian R, Laird N. Meta-analysis in clinical trials. *Control Clin Trials*. 1986;7(3):177-188. doi:10.1016/0197-2456(86)90046-2

3. Schuemie MJ, Hripcsak G, Ryan PB, Madigan D, Suchard MA. Empirical confidence interval calibration for population-level effect estimation studies in observational healthcare data. *Proc Natl Acad Sci U S A*. 2018;115(11):2571-2577. doi:10.1073/pnas.1708282114

4. Schuemie MJ, Ryan PB, DuMouchel W, Suchard MA, Madigan D. Interpreting observational studies: why empirical calibration is needed to correct p‐values. *Stat Med*. 2014;33(2):209-218. doi:10.1002/sim.5925

5. Schuemie MJ, Hripcsak G, Ryan PB, Madigan D, Suchard MA. Robust empirical calibration of p‐values using observational data. *Stat Med*. 2016;35(22):3883-3888. doi:10.1002/sim.6977

6. Voss, Boyce, Ryan, van der Lei, Rijnbeek, Schuemie. Accuracy of an automated knowledge base for identifying drug adverse reactions. *Journal of Biomedical Informatics*. 2017;66:72-81. doi:10.1016/j.jbi.2016.12.005

7. Winnenburg, Sorbello, Ripple, et al. Leveraging MEDLINE indexing for pharmacovigilance – Inherent limitations and mitigation strategies. *Journal of Biomedical Informatics*. 2015;57:425-435. doi:10.1016/j.jbi.2015.08.022

8. Duke J, Friedlin J, Li X. Consistency in the safety labeling of bioequivalent medications. *Pharmacoepidemiol Drug Saf*. 2013;22(3):294-301. doi:10.1002/pds.3351

9. Evans SJW, Waller PC, Davis S. Use of proportional reporting ratios (PRRs) for signal generation from spontaneous adverse drug reaction reports. *Pharmacoepidemiol Drug Saf*. 2001;10(6):483-486. doi:10.1002/pds.677

10. Banda Juan M., Evans Lee, Vanguri Rami S., Tatonetti Nicholas P., Ryan Patrick B., Shah Nigam H. A curated and standardized adverse drug event resource to accelerate drug safety research. *Sci Data*. 2016;3:160026. doi:10.1038/sdata.2016.26

**Text S2.** Data source descriptions

**MDCR**

Merative MarketScan Medicare Supplemental and Coordination of Benefits Database (MDCR) represents health services of retirees in the United States with primary or Medicare supplemental coverage through privately insured fee-for-service, point-of-service, or capitated health plans. These data include adjudicated health insurance claims (e.g. inpatient, outpatient, and outpatient pharmacy). Additionally, it captures laboratory tests for a subset of the covered lives.

**MDCD**

Merative MarketScan Multi-State Medicaid Database (MDCD) contains adjudicated US health insurance claims for Medicaid enrollees from multiple states and includes hospital discharge diagnoses, outpatient diagnoses and procedures, and outpatient pharmacy claims as well as ethnicity and Medicare eligibility. Members maintain their same identifier even if they leave the system for a brief period; however the dataset lacks lab data.

**JMDC**

Japan Medical Data Center (JMDC) database consists of data from 60 society-managed health insurance plans covering workers aged 18 to 65 and their dependents (children younger than 18 years old and elderly people older than 65 years old). JMDC data includes membership status of the insured people and claims data provided by insurers under contract (e.g. patient-level demographic information, inpatient and outpatient data inclusive of diagnosis and procedures, and prescriptions as dispensed claims information). Claims data are derived from monthly claims issued by clinics, hospitals and community pharmacies; for claims only the month and year are provided however prescriptions, procedures, admission, discharge, and start of medical care as associated with a full date. All diagnoses are coded using ICD-10. All prescriptions refer to national Japanese drug codes, which have been linked to ATC. Procedures are encoded using local procedure codes, which the vendor has mapped to ICD-9 procedure codes. The annual health checkups report a standard battery of measurements (e.g. BMI), which are not coded but clearly described.

**NHIS/NSC**

Korea National Health Insurance Service / National Sample Cohort (NHIS/NSC) is the national administrative claims database covering the South Korea population. It contains a 2% population sample cohort from 2002 - 2013.

**PanTher**

Optum Pan-Therapeutic (PanTher) is an aggregated electronic health record repository from US health systems and contains Humedica’s electronic health record data. The medical record data includes clinical information, inclusive of prescriptions as prescribed and administered, lab results, vital signs, body measurements, diagnoses, procedures, and information derived from clinical notes using natural language processing (NLP).

**IMSG**

IMS/IQVIA Disease Analyzer Germany (IMSG) contains electronic health records data from German practices. The data are collected from physician practices and medical centers for all ages. Mostly primary care physician data however some data from specialty practices (where practices are electronically connected to each other) and some lab data is included. Key attributes include demographics, prescriptions as prescribed at brand level, diagnosis, lab measurements, actions (e.g. referrals, sick notes).

**CUMC**

Columbia University Medical Center (CUMC) is an electronic health record consisting of over 5 million patients from the New York-Presbyterian hospital and affiliated academic physician practice in New York.

**Text S3**. Brief summary of the Observational Medical Outcomes Partnership (OMOP) Common Data Model (CDM), standardized terminologies, and the Health Data Analytics-to-Evidence Suite (HADES)

The Observational Health Data Sciences and Informatics (OHDSI) initiative is a public-private collaboration aimed at advancing active drug safety surveillance through robust scientific methods and data infrastructure. OHDSI conducts systematic research to address key methodological and technological requirements for a national medical product safety system, ultimately seeking to enhance the safety and effectiveness of pharmaceuticals using observational data.

OHDSI has developed the Observational Medical Outcomes Partnership (OMOP) Common Data Model (CDM) to standardize data structures across diverse healthcare databases without altering the original data content.^6,7^ This CDM is adaptable to both electronic health record (EHR) and administrative claims data, allowing organizations, data venders, and data users to convert uniquely formatted data sources into a consistent structure using a unified vocabulary. Key coding systems, such as ICD-9-CM, ICD-10-CM, CPT-4, SNOMED, and LOINC, are integrated within this CDM, enabling comparable analyses across multiple data sources. The model facilitates uniform application of methods, tools, and benchmarks, enhancing cross-database interpretability. OHDSI developed the Health Data Analytics-to-Evidence Suite (HADES) software to support evidence generation, influencing both clinical decision-making and regulatory policies.^9^ This open-source software suite provides tools for characterization, causal inference, and patient-level prediction, facilitating observational research across the OHDSI data network.

Quality assurance tools support the data transformation process and validate key population characteristics within each database. To ensure semantic consistency, OMOP developed standardized vocabulary mappings to harmonize clinical terminologies, bridging codes like ICD-9-CM to SNOMED-CT and MedDRA.^8^ These mappings were based on empirical evaluations of data retention, semantic consistency, outcome prevalence, and reliability in detecting drug-outcome associations across databases. Overall, SNOMED-CT and MedDRA were confirmed as suitable standardized vocabularies for active medical surveillance, ensuring reliability and comparability across large, disparate datasets in observational health research and justifying their use as the standard vocabularies of the OMOP CDM.

| **Minimally-detectable risk-ratio (MDRR) \| Threshold: 10** |
| --- |
| 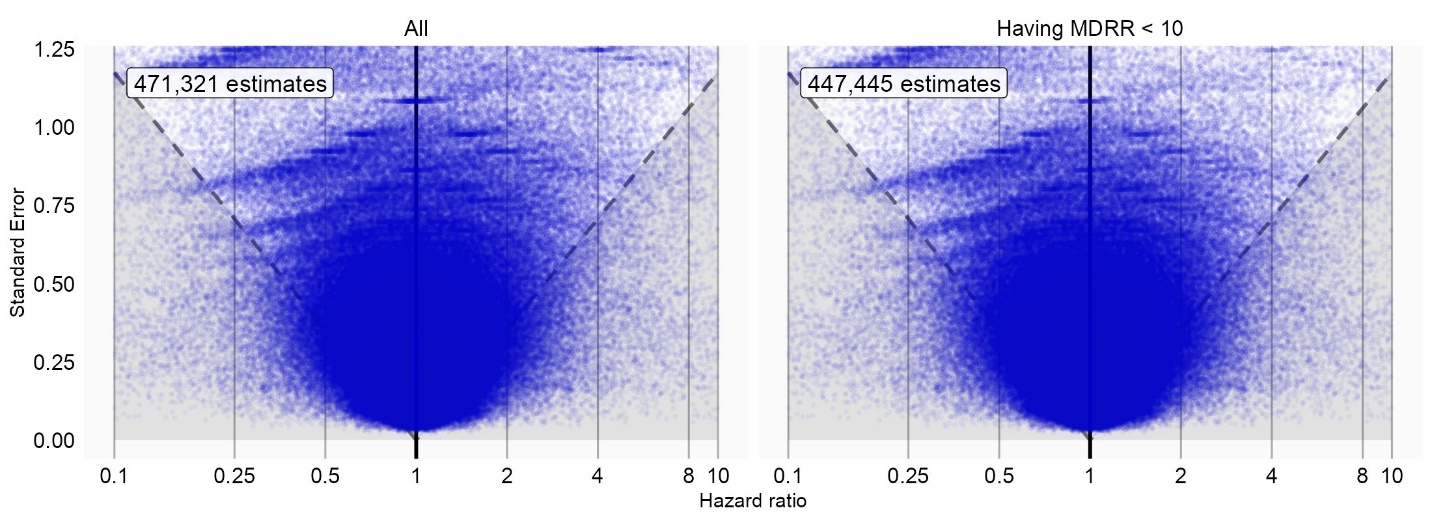 |
| 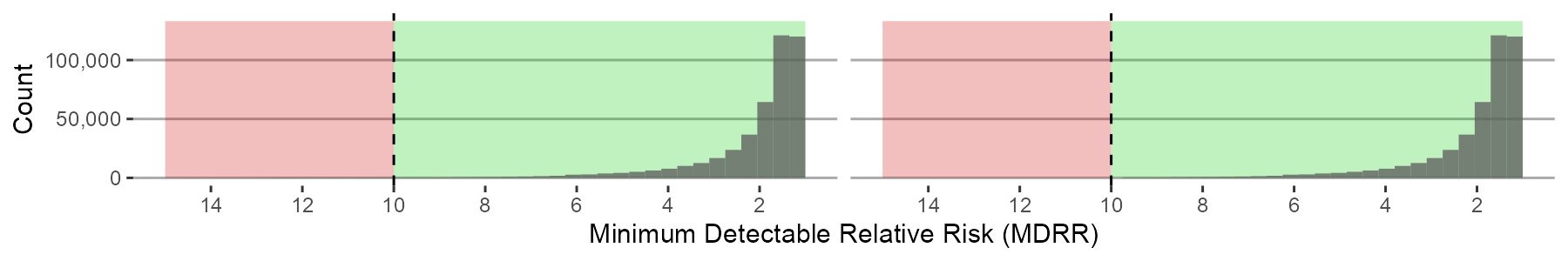 |
| **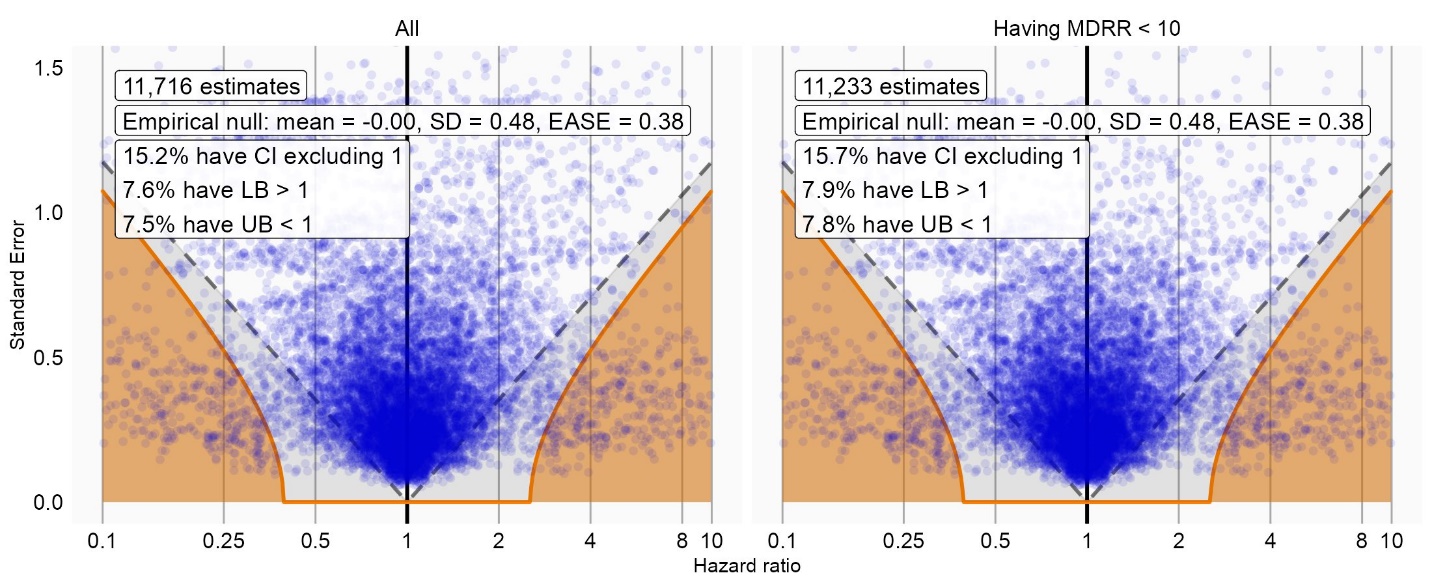** |

**Figure S1**. Top panel: Effect estimates plotted against standard errors for the full set of LEGEND studies (left) and those with MDRR<10 (right). Middle panel: The distribution of the minimum detectable relative risk (MDRR) statistic for all negative control studies (left) and those with MDRR<10 (right). Bottom panel: The empirical null distribution for all negative control studies (left) and those with MDRR<10 (right).

| **Empirical Equipoise \| Threshold: 0.50** |
| --- |
| 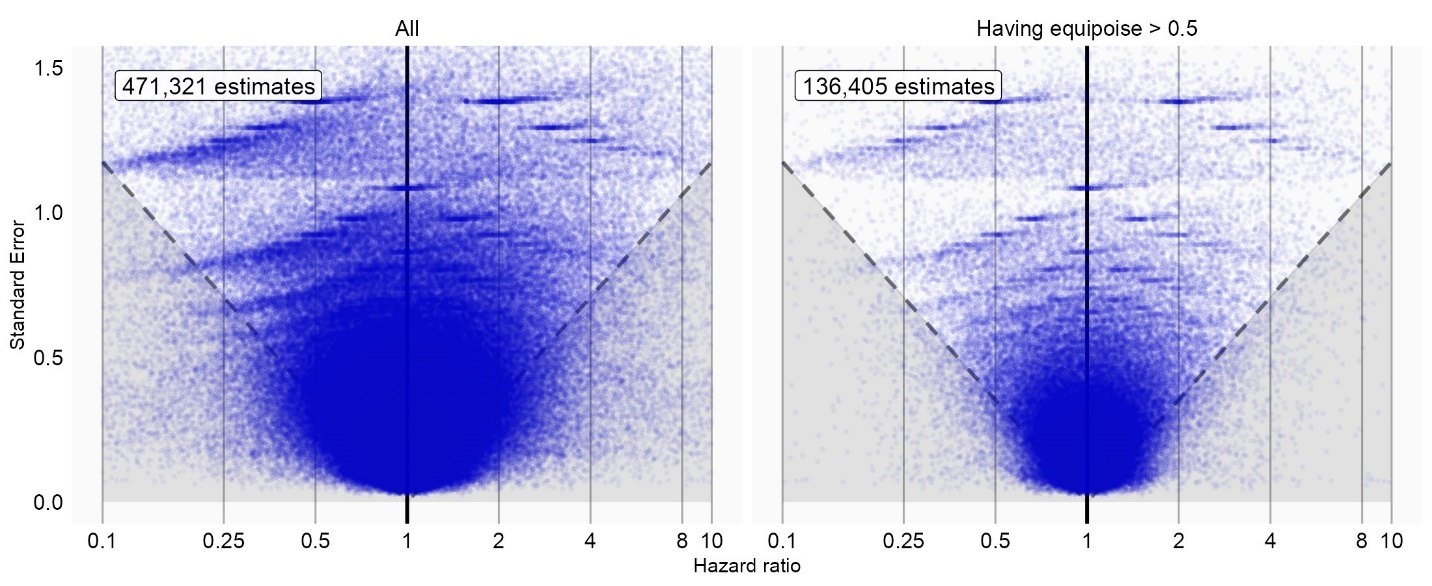 |
| 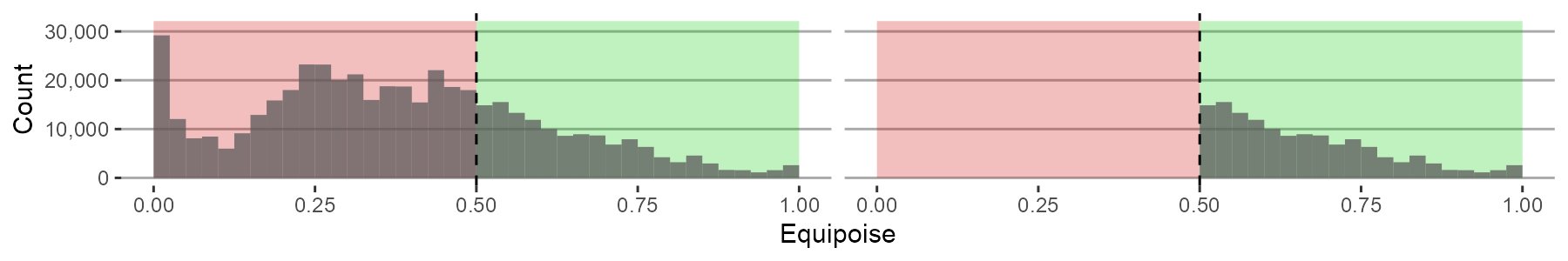 |
| 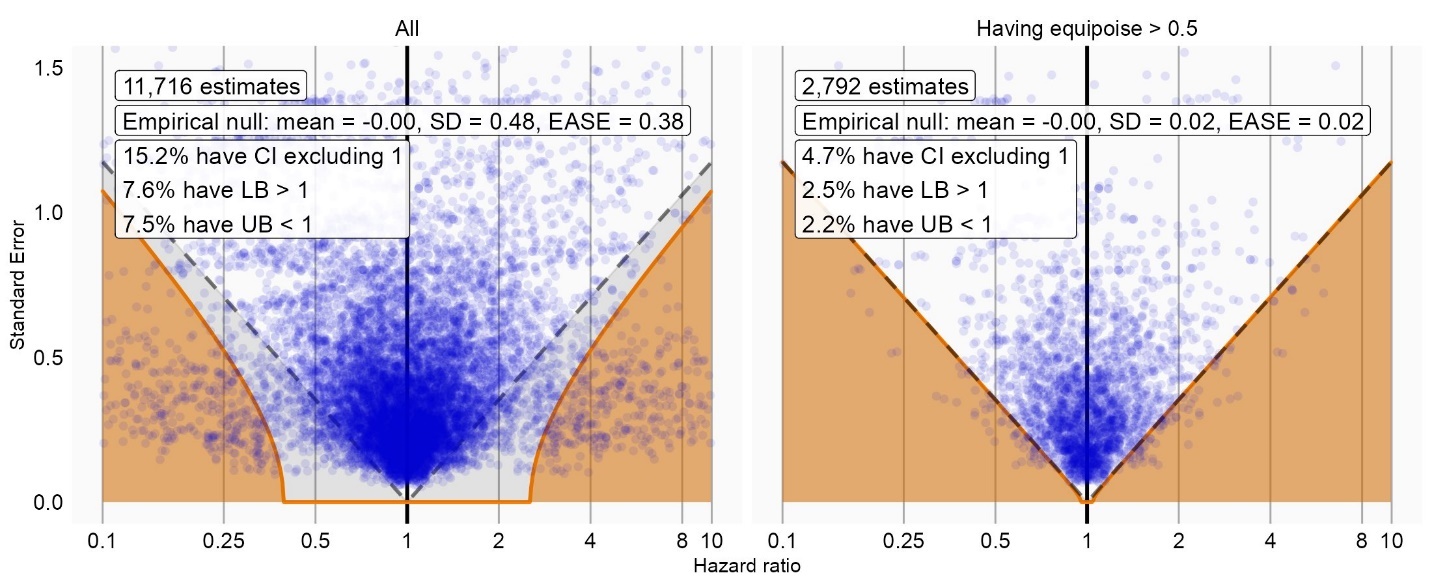 |

**Figure S2**. Top panel: Effect estimates plotted against standard errors for the full set of LEGEND studies (left) and those with equipoise<0.5 (right). Middle panel: The distribution of the proportion of patients in equipoise across all negative control studies (left) and those with equipoise<0.5 (right). Bottom panel: The empirical null distribution for all negative control studies (left) and those with equipoise<0.5 (right).

| **Empirical equipoise \| Threshold: 0.10** |
| --- |
| 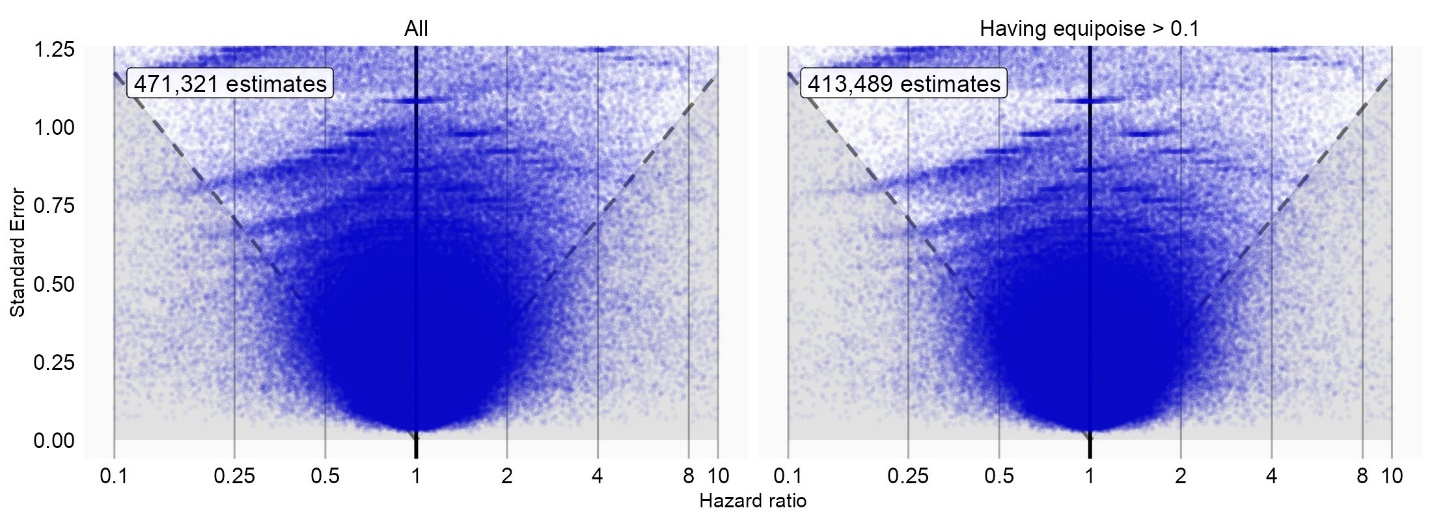 |
| 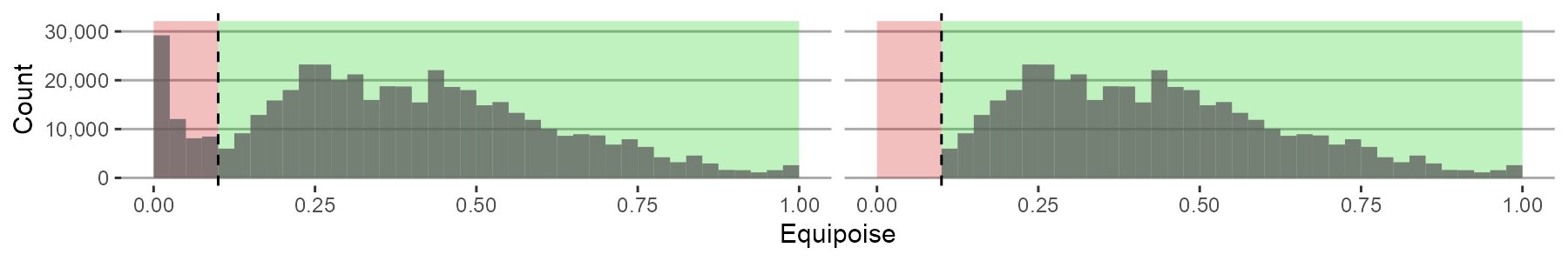 |
| 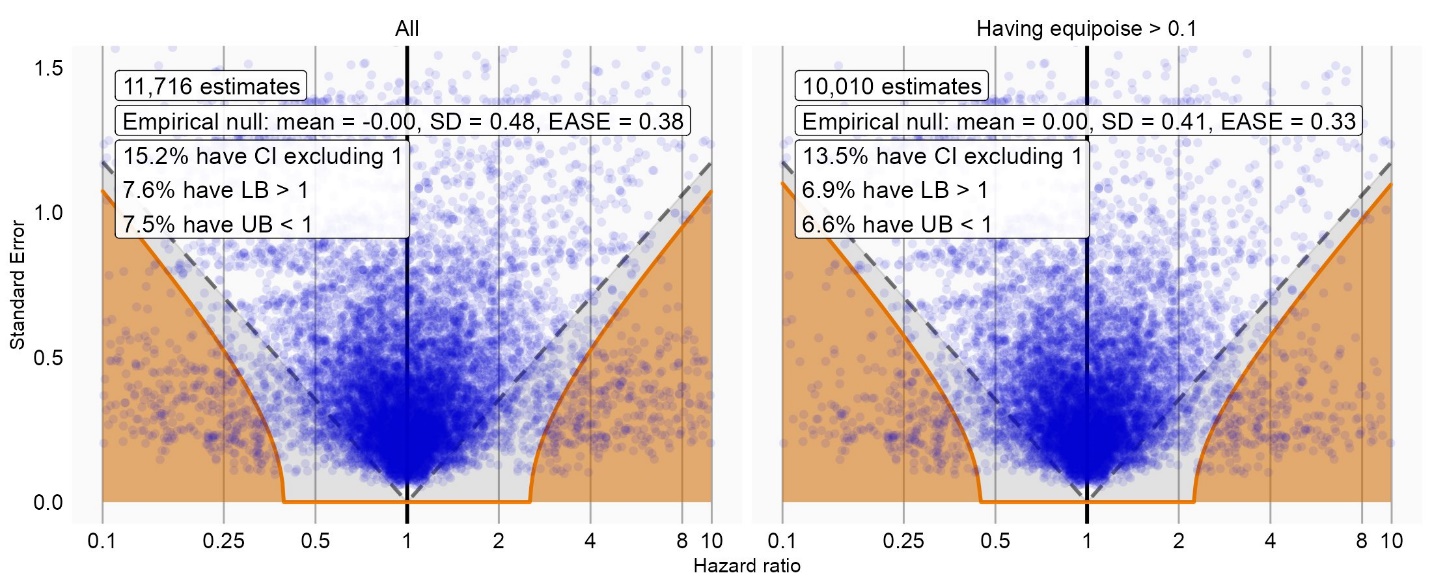 |

**Figure S3**. Top panel: Effect estimates plotted against standard errors for the full set of LEGEND studies (left) and those with equipoise<0.1 (right). Middle panel: The distribution of the proportion of patients in equipoise across all negative control studies (left) and those with equipoise<0.1 (right). Bottom panel: The empirical null distribution for all negative control studies (left) and those with equipoise<0.1 (right).

| **Generalizability SDM \| Threshold: 0.25** |
| --- |
| 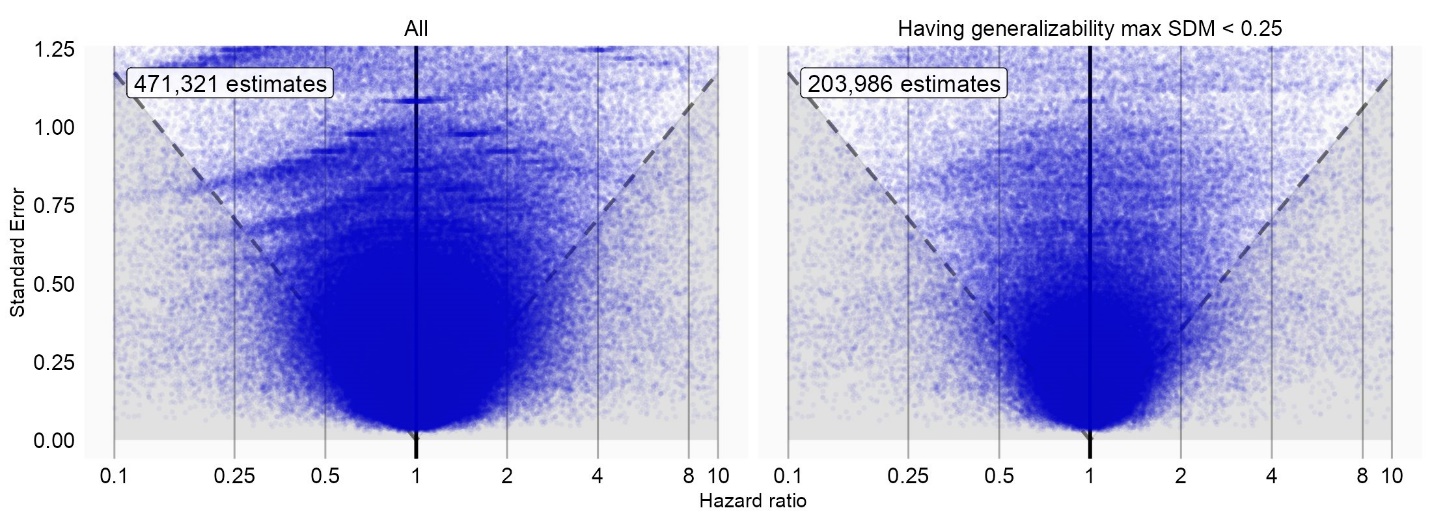 |
| 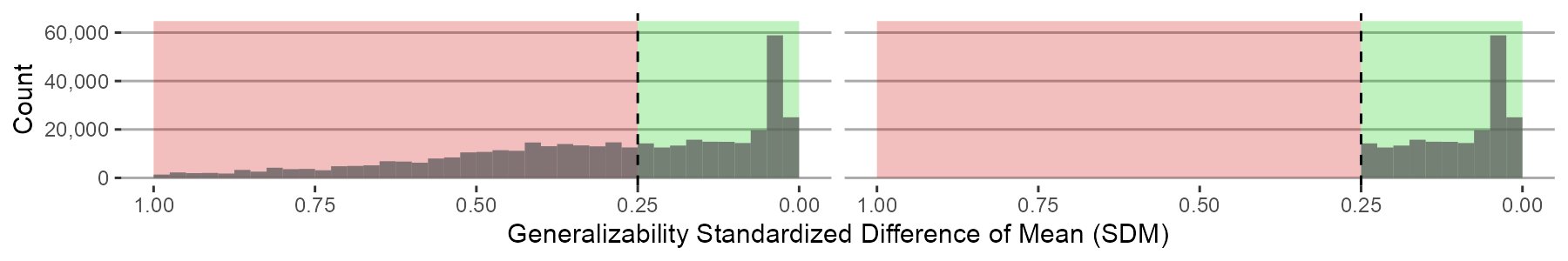 |
| 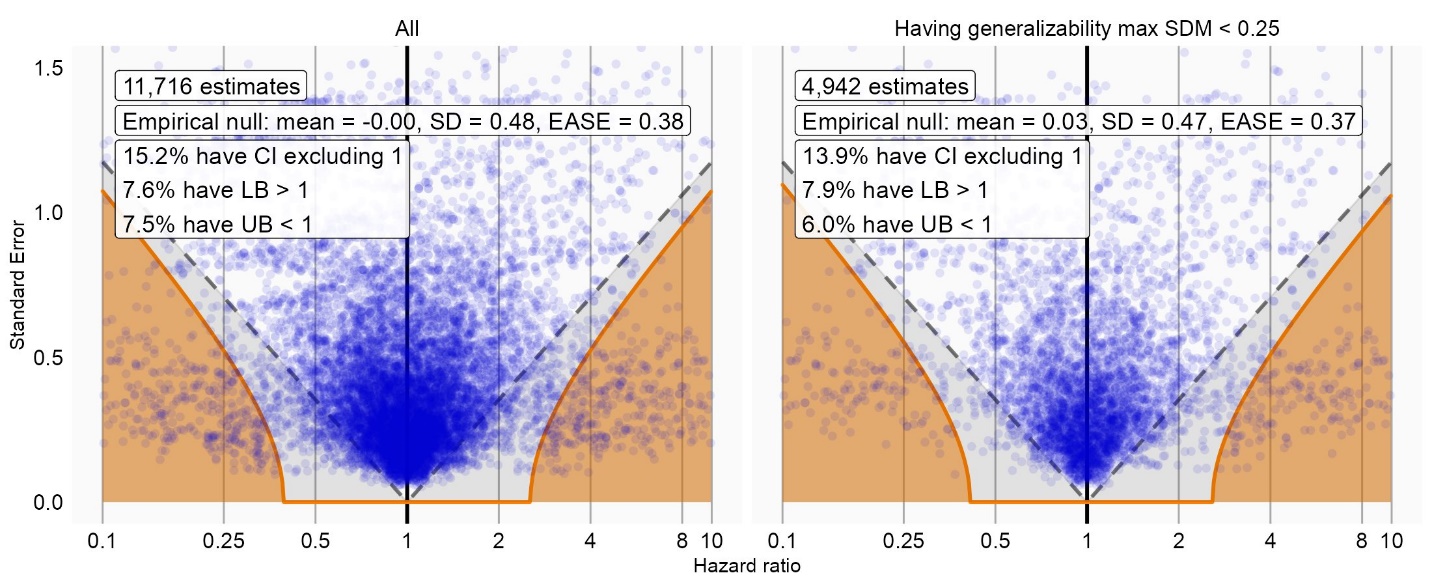 |

**Figure S4**. Top panel: Effect estimates plotted against standard errors for the full set of LEGEND studies (left) and those with generalizability SDM<0.25 (right). Middle panel: The distribution of the generalizability standardized difference of means (SDM) for all negative control studies (left) and those with generalizability SDM<0.25 (right). Bottom panel: The empirical null distribution for all negative control studies (left) and those with generalizability SDM<0.25 (right).

| **Expected Absolute Systematic Error (EASE) \| Threshold: 0.25** |
| --- |
| 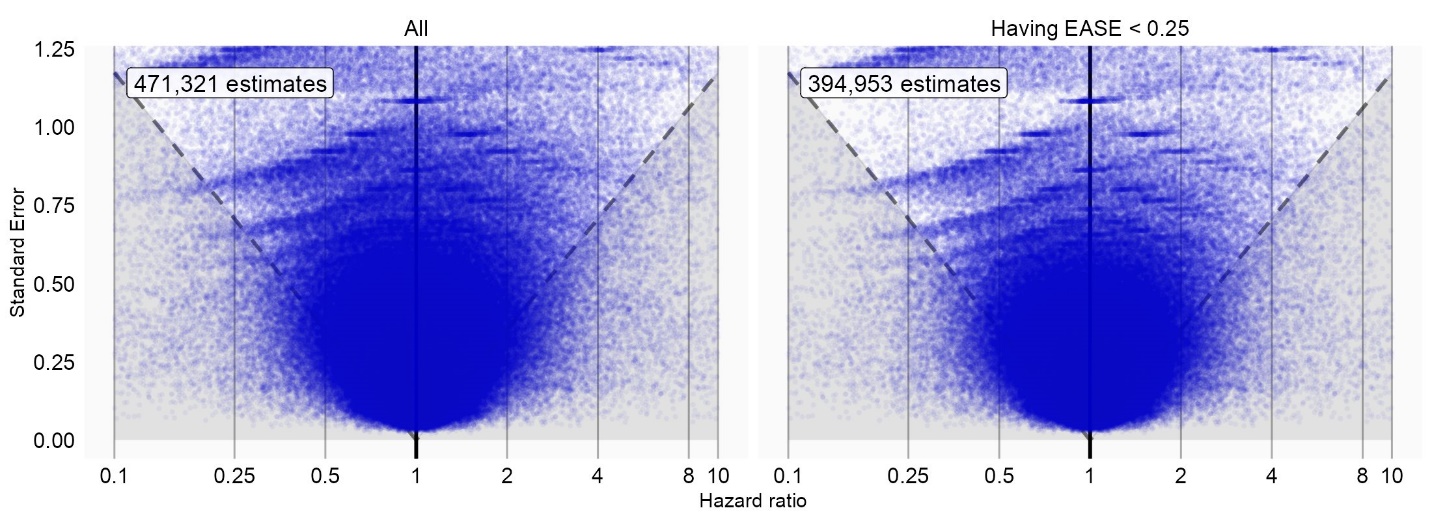 |
| 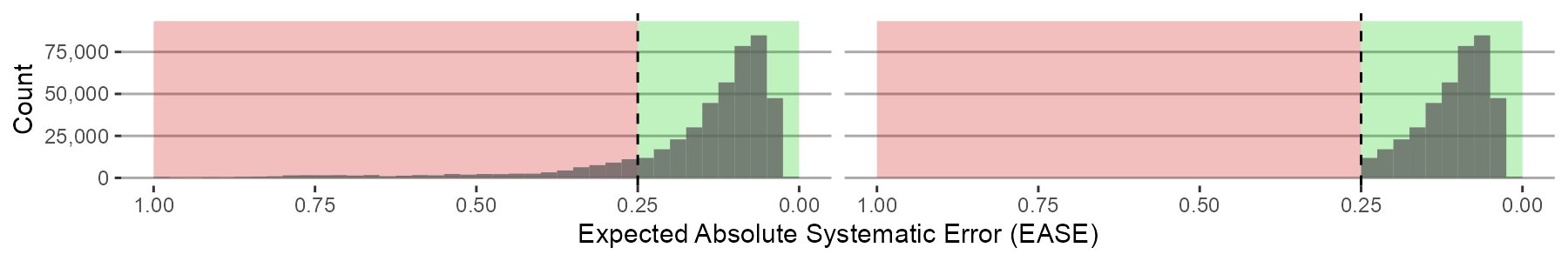 |
| 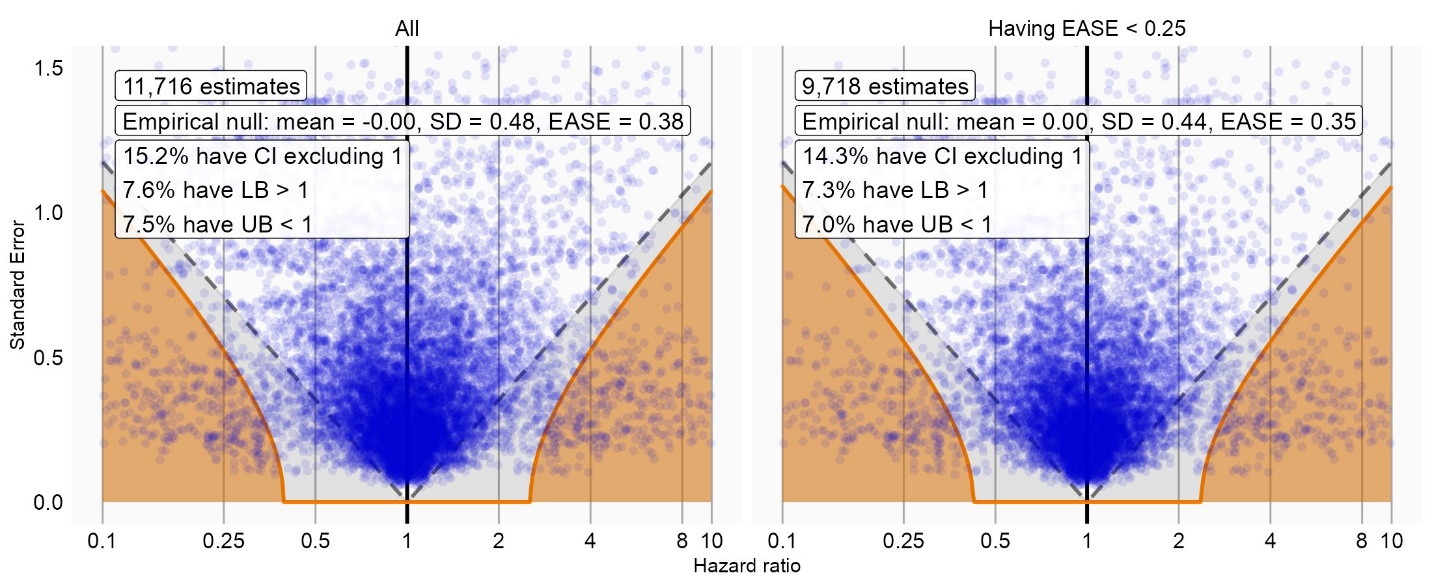 |

**Figure S5**. Top panel: Effect estimates plotted against standard errors for the full set of LEGEND studies (left) and those with EASE<0.25 (right). Middle panel: The distribution of the expected absolute systematic error (EASE) for all negative control studies (left) and those with generalizability EASE<0.25 (right). Bottom panel: The empirical null distribution for all negative control studies (left) and those with EASE<0.25 (right).

| 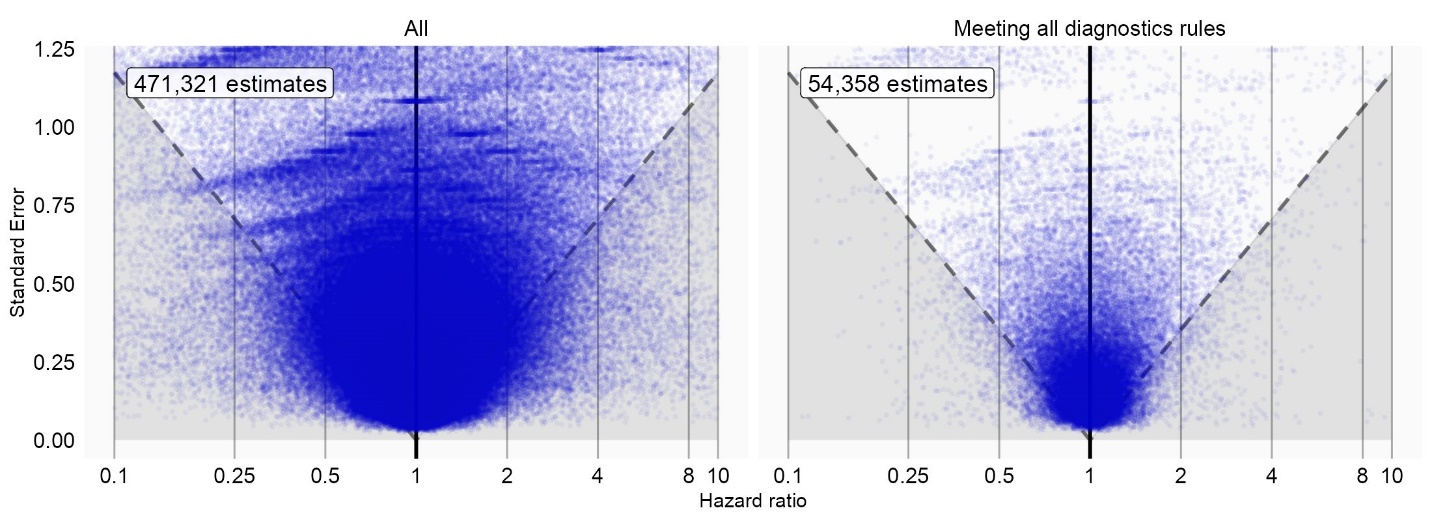 |
| --- |
| 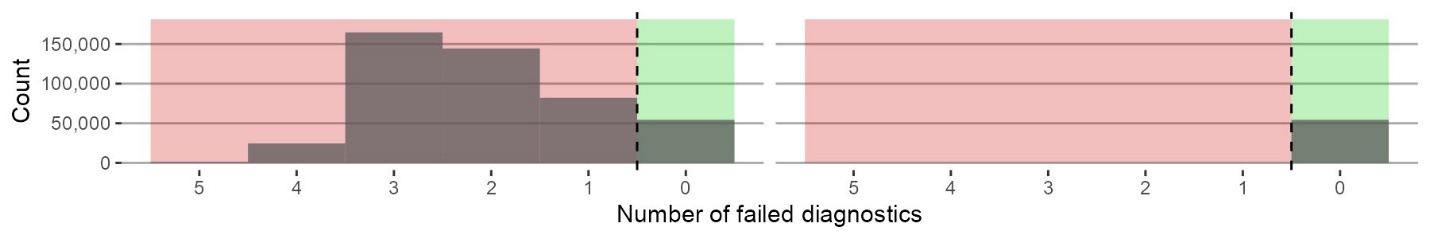 |
| 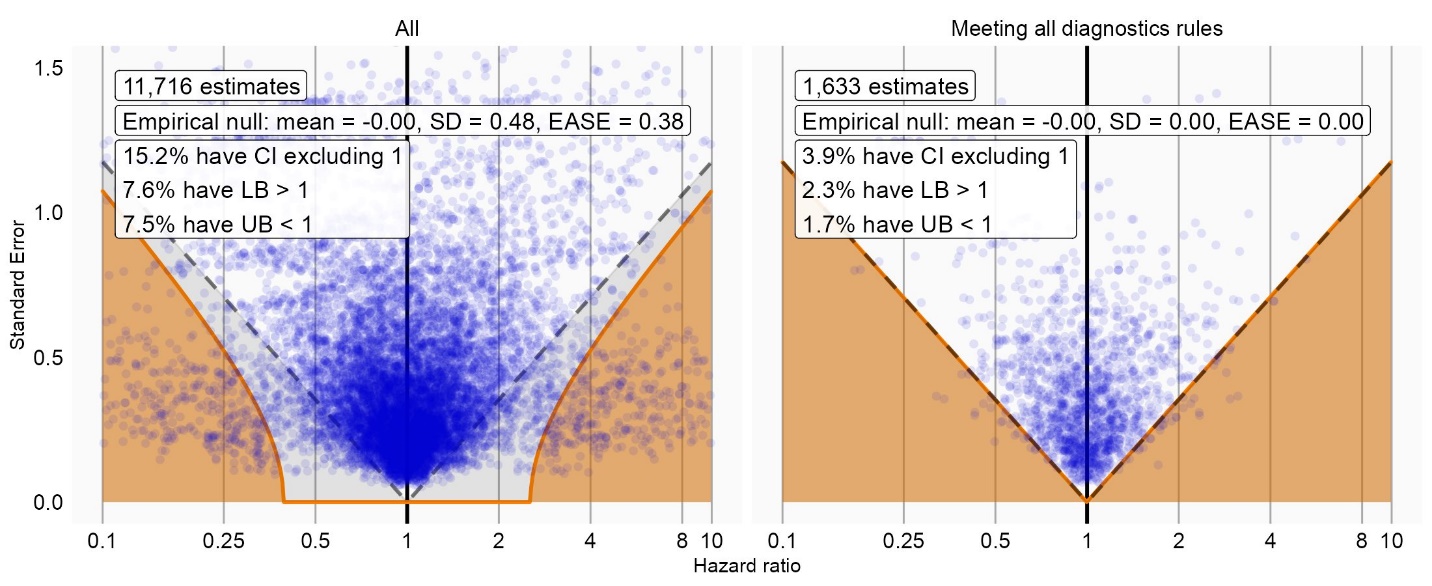 |

**Figure S6**. Top panel: Effect estimates plotted against standard errors for the full set of LEGEND studies (left) and the subset of LEGEND studies that satisfied all diagnostic criteria (right). Middle panel: The distribution of number of failed diagnostics for all negative control studies (left) and those which satisfied all diagnostic criteria (right). Bottom panel: The empirical null distribution for all negative control studies (left) and those which satisfied all diagnostic criteria (right).
